# The Crystal Structures of Dystrophin and Utrophin Spectrin Repeats: Implications for Domain Boundaries

**DOI:** 10.1371/journal.pone.0040066

**Published:** 2012-07-20

**Authors:** Muralidharan Muthu, Kylie A. Richardson, Andrew J. Sutherland-Smith

**Affiliations:** Institute of Molecular BioSciences, Massey University, Palmerston North, New Zealand; University of Minnesota, United States of America

## Abstract

Dystrophin and utrophin link the F-actin cytoskeleton to the cell membrane via an associated glycoprotein complex. This functionality results from their domain organization having an N-terminal actin-binding domain followed by multiple spectrin-repeat domains and then C-terminal protein-binding motifs. Therapeutic strategies to replace defective dystrophin with utrophin in patients with Duchenne muscular dystrophy require full-characterization of both these proteins to assess their degree of structural and functional equivalence. Here the high resolution structures of the first spectrin repeats (N-terminal repeat 1) from both dystrophin and utrophin have been determined by x-ray crystallography. The repeat structures both display a three-helix bundle fold very similar to one another and to homologous domains from spectrin, α-actinin and plectin. The utrophin and dystrophin repeat structures reveal the relationship between the structural domain and the canonical spectrin repeat domain sequence motif, showing the compact structural domain of spectrin repeat one to be extended at the C-terminus relative to its previously defined sequence repeat. These structures explain previous *in vitro* biochemical studies in which extending dystrophin spectrin repeat domain length leads to increased protein stability. Furthermore we show that the first dystrophin and utrophin spectrin repeats have no affinity for F-actin in the absence of other domains.

## Introduction

The X-linked Duchenne and Becker muscular dystrophies (DMD and BMD) are caused by mutations in dystrophin (Dys) associated with muscle plasma membrane fragility, increased intracellular Ca^2+^ levels and proteolytic activity [1], [2] leading to muscle myofibrillar decomposition with subsequent replacement by fibrous and fat tissue. Dystrophin is a large (427 kD) multidomain protein expressed in skeletal and cardiac muscle where it binds to the dystrophin-associated glycoprotein (DAG) complex of the plasma membrane and to F-actin connecting the cytoskeleton to the membrane. Utrophin (Utr), the 395 kD autosomal homologue of Dys [3] is widely expressed in non muscle tissues (reviewed [2]) but is restricted to the myotendinous and neuromuscular junctions of differentiated muscle [4] and is located at the sarcolemma of developing fetal muscle [5]. Dys and Utr are members of the spectrin-like family of actin-binding proteins, which also includes the eponymous spectrin and α-actinin F-actin cross-linking proteins, based on their domain organisation: i) An N-terminal actin-binding domain (ABD) consisting of two calponin homology domains that bind F-actin [6] and keratin 9/18 [7] with micromolar affinities. ii) An elongated central rod region consisting of multiple (22 for Utr, 24 for Dys) ∼105 amino acid spectrin repeat domains, interrupted by two hinge regions. iii) The C-terminal region containing a WW domain, EF hands, a cysteine-rich domain and a coiled-coiled motif. The C-terminal domains interact with the DAG complex that spans the plasma membrane to the extracellular matrix and also, via adaptor proteins dystrobrevin and syntrophin, nitric oxide synthase (NOS), erbB-4 receptor protein kinase and voltage-gated sodium channels [2]. Utr and Dys are important for stabilizing this elaborate plasma membrane assembly and its interactions with the extracellular matrix [8] forming a strong mechanical link from the actin cytoskeleton to the plasma membrane [9]. Dys and Utr potentially act as cellular biomechanical shock-absorbers limiting damage to the plasma membrane with the spectrin repeat domains acting as spring units by undergoing force-induced unfolding [10]. The importance of individual spectrin repeat domains for overall Dys function was initially assumed to be low owing to the observation that in-frame deletion of repeats 4–19, (nearly half of Dys) gave rise to a mild BMD phenotype [11]. However this assumption is now becoming increasingly challenged; e.g. an in-frame two amino acid deletion that destabilizes repeat 23 is associated with a severe DMD phenotype [12].

The Dys/Utr spectrin repeat domains, and their underlying heptad repeat sequence motif, can be identified by sequence alignment although the homology between the repeats is much lower than between the repeats from α/β-spectrin [13], [14]. For α-actinin and α/β-spectrin the repeat domains are involved in anti-parallel dimerisation (reviewed [15]) that does not occur for dystrophin [16]. The structure and the domain boundaries of the spectrin repeats within the Dys/Utr central rod region, with respect to the repeating sequence motif, remained unclear in the absence of structural data with alternative models existing [17], [18], [19], [20], [21]. One model proposes that each spectrin repeat is a self-contained domain structure connected via its C-terminus to the N-terminus of the next domain via a continuous helix. A second model proposes that the repeats are structurally nested such that the N-terminal helix A (or part of) from repeat ‘n’ is juxtaposed structurally with the previous (n−1) spectrin repeat domain. Owing to the sequence diversity of the Dys/Utr repeats their organisation throughout the protein may not necessarily be uniform so a mixture of these models remains possible. Single and double dystrophin spectrin repeats constructs can have quite different folding properties [22] with their stability depending on repeat flanking sequence length [21]. The structural organization of the repeat domains has important implications for protein function; for Dys and Utr to act in a shock absorbing role, force-induced unfolding of the central rod region could occur via a domain based mechanism (in concert with the hinge regions) or via a more cooperative mechanism for a nested repeat domain arrangement. Spectrin repeats provide sites for protein-protein interactions; Dys repeats 11–17 bind F-actin [23] in contrast to Utr where repeats 1–10 augment F-actin binding by the ABD [24]. The different binding modes of Dys and Utr lead to differences in F-actin rigidity and structural dynamics [25]. The intermediate filament protein synemin binds the Dys central rod region at two sites; one within repeats 1–5 and the other 11–14 [26]. Dys/Utr repeats 8 and 9 interact with Par-1 kinase required for the stable association with β-dystroglycan [27]. Transfection of Dys cDNA containing repeats 16 and 17 targets neuronal NOS to the muscle plasma membrane ameliorating dystrophic symptoms in the *mdx* (dystrophin null) mouse [28]. In addition to protein partners, Dys constructs containing SR1–3 and SR4–19 have phospholipid binding activity [29], [30]. These interaction studies highlight functions for particular Dys/Utr spectrin repeats in addition to a more general role as biomechanical response domains.

Spectrin repeat structures from α/β−spectrin [31], [32], [33], [34], [35], α-actinin [36], [37] and plakins [38], [39], [40] have a three-helix (A–B–C) bundle fold with the multiple repeat structures showing that the C helix of the preceding repeat is continuous with the A’ helix of the next. The structural nature of this connecting linker between repeats has important implications for the biophysical characteristics of the whole rod region [41].

Considerable interest in Utr has been stimulated by the possibility of over-expressing Utr in patients afflicted with DMD and thereby replacing the deficient or defective Dys with a non-immunogenic functional homologue. Studies showing that an *Utr* transgene alleviates the disease symptoms in the DMD *mdx* mouse model [42] have provided encouragement for approaches using pharmacological [43] and artificial transcription factor [44] *Utr* upregulation strategies. Structural and functional clarification of the molecular roles and properties of Dys/Utr spectrin repeats, and specifically an improved understanding of the nature of the linkers joining the repeats [45], is becoming increasingly important as efforts intensify to explore the use of shortened mini/micro *Dys*/*Utr* transgenes (omitting specific spectrin repeats) in a clinical context [42]. For example it remains unclear as to why a micro-dystrophin with repeats 4–23 deleted, removing the actin binding activity within the central rod region, can effectively ameliorate the dystrophic phenotype in the *mdx* mouse but constructs with the entire rod region deleted (Δrepeats 1–24) cannot [46].

Estimating the potential for therapeutic replacement of Dys with Utr requires their structural and functional characterization, which by virtue of their large sizes is well suited to a domain dissection approach. We present the first structures of Dys and Utr spectrin repeat domains that reveal the phasing of the helices with respect to the sequence motif and its underlying heptad repeat. We also show that the Utr and Dys spectrin repeat domains are largely equivalent although differences can exist in the arrangement of a conserved hydrophobic stacking interaction within the helical bundle core revealing a degree of structural plasticity. It had previously been observed that a Dys repeat 2 construct extended to comprise 119 residues showed greater stability than shorter domains leading to the hypothesis that the minimum-folding domain extended into the adjoining sequence repeat, though it was unclear quite how long such an extension would be [18]. We now show the Dys and Utr spectrin repeat domain structures with respect to their sequence repeats that will aid the design of further constructs for both functional studies and future therapeutic mini-transgene strategies.

## Results

### The Crystal Structures of the N-terminal (Repeat 1) Spectrin Repeats from Utrophin and from Dystrophin

We have determined high resolution structures of the N-terminal spectrin repeats (repeat 1) from Utr and from Dys. The Utr N-terminal spectrin repeat 1 (Utr-SR1) crystallised with two molecules in the asymmetric unit of the P2_1_2_1_2_1_ cell and has been refined to a final model with crystallographic R and R_free_ values of 0.200 and 0.235 with diffraction data of outer limit 1.95 Å resolution (table 1). The final model (PDB: 3UUL) is of good geometry containing residues 310–424 (monomer A), 308–424 (monomer B) and 206 water molecules. 98.7% of amino acids are in the preferred regions of the Ramachandran plot, 1.3% (2 residues) in the allowed region and none in the disallowed region as defined by MOLPROBITY [47]. Additionally we have determined the structure of a six amino acid longer version of the Utr-SR1 protein (amino acids 308–430, Utr-SR1-L) produced as a result of presumed proteolysis during crystallization experiments of an Utr N-terminal two spectrin repeat protein construct (Utr-SR1-SR2; 308–537). SDS-PAGE analysis of dissolved Utr-SR1-L crystals showed they contained a single 15 kD species. The Utr-SR1-L crystals are isomorphous with Utr-SR1 with insufficient space in the crystal lattice for the complete two domain SR1–SR2 structure. The Utr-SR1-L C-terminus is defined at 430 the last residue with interpretable electron density and has been refined to R of 0.201 and R_free_ 0.268 against x-ray diffraction data with a high resolution limit of 2.0 Å (PDB: 3UUM). The Dys N-terminal (repeat 1) spectrin repeat (Dys-SR1) also crystallized with two molecules in the asymmetric unit but there is no relationship between the two crystal symmetries. Dys-SR1 was refined to yield a model containing residues 338–453 (monomer A), 339–452 (monomer B) with R of 0.193 and R_free_ 0.260 against x-ray diffraction data with a high resolution limit of 2.3 Å (PDB: 3UUN). The Ramachandran plot shows 98.7% of residues in preferred regions with two residues in an allowed region.

**Table 1:** Crystallographic x-ray data collection and refinement statistics.

Protein	Utr-SR1	Utr-SR1-L	Dys-SR1
Space group	P2_1_2_1_2_1_	P2_1_2_1_2_1_	P3_2_21
Cell dimensions	a = 43.00 Å	a = 42.74 Å	a = b = 76.04 Å
	b = 58.66 Å	b = 58.02 Å	c = 66.49 Å
	c = 91.45 Å	c = 91.27 Å	α = β = 90°
	α = β = γ = 90°	α = β = γ = 90°	γ = 120°
Resolution range (Å)	35.92–1.95	35.87–2.00	33.24–2.30
Resolution outer shell (Å)	2.06–1.95	2.11–2.00	2.42–2.30
Unique reflections	17305	15608	9999
Multiplicity (outer shell)	4.0 (3.9)	5.7 (5.5)	7.3 (7.4)
Completeness (outershell) (%)	99.9 (100)	98.4 (97.3)	98.4 (97.4)
R_merge_ (outer shell)	0.085 (0.406)	0.141 (0.720)	0.113 (0.605)
Mean I/sigI (outer shell)	10.2 (3.0)	7.6 (2.3)	11.7 (3.1)
Mosaicity (°)	0.91	1.55	0.64
Number of reflections	16320	14759	9035
Number of protein atoms	1857	1988	1866
R-factor_work_	0.200	0.201	0.193
R-factor_free_	0.235	0.268	0.260
Rmsd from ideal geometry:			
Bond lengths (Å)	0.015	0.019	0.017
Bond angles (^o^)	1.24	1.59	1.61
Average B-factor (Å^2^)	37.0	37.4	43.4
Wilson B-factor (Å^2^)	23.1	26.1	35.9
Ramachandran plot:			
Most favoured region (%)	98.7	99.2	98.7
Outliers	0.0	0.0	0.0

R_merge_ = Σ|I_i_−<I_i_>|/ΣI_i_ where I_i_ is the intensity of a single reflection and <I_i_> is the mean intensity of that reflection.

R_working_ = Σ|F_o_−F_c_|/ΣF_o_ R_free_ is the R-factor calculated for the cross-validated test set of reflections.

**Figure 1:**
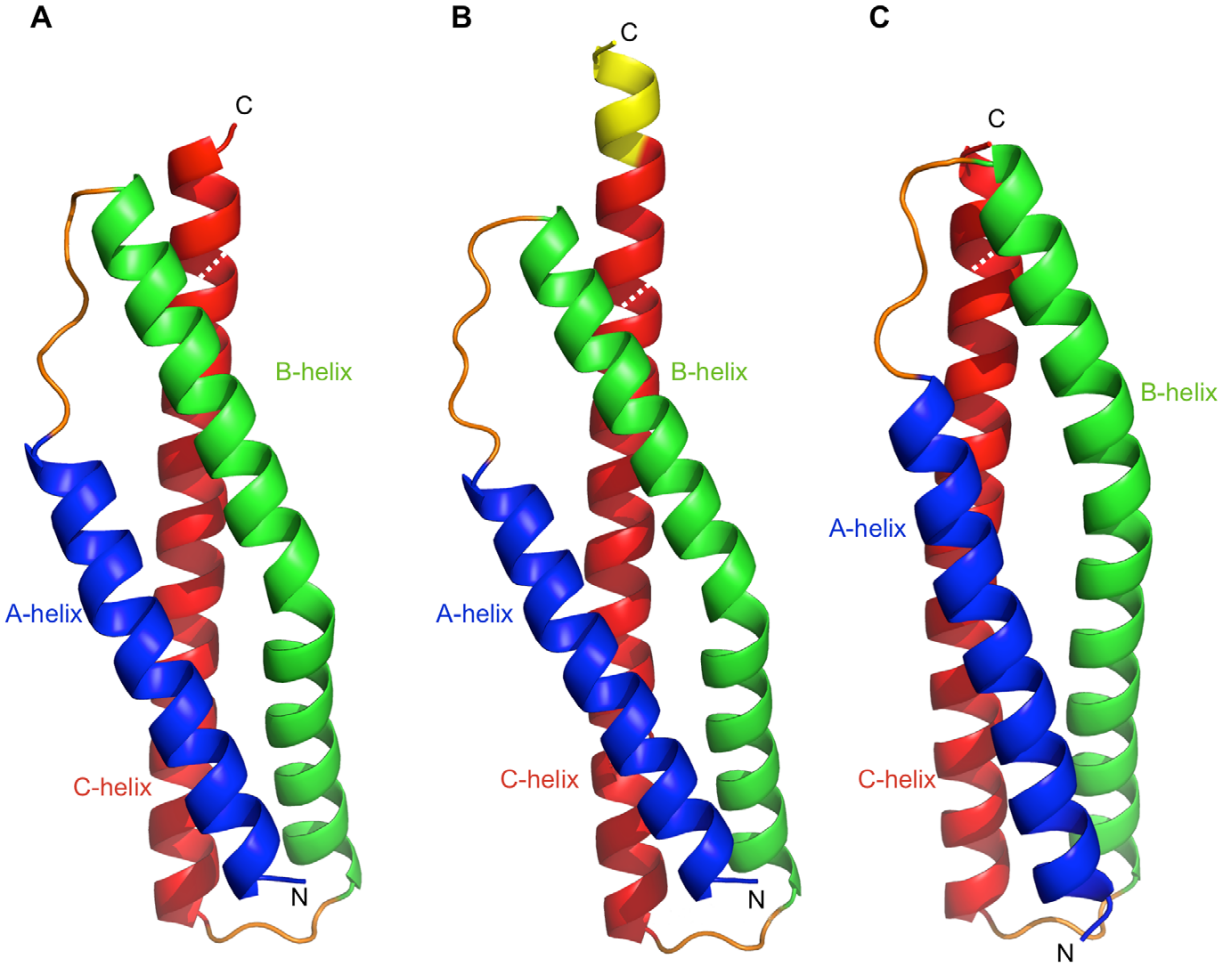
Utrophin and dystrophin spectrin repeat one crystal structures. Cartoon representations of A) Utr-SR1, B) Utr-SR1-L and C) Dys-SR1 structures colour-coded helix A blue, helix B green, helix C red and A–B and B–C loops in orange. The C-terminal extension of Utr-SR1-L is coloured yellow and the position of the sequence-defined repeat C-terminus is shown with a dashed white line.

**Figure 2:**
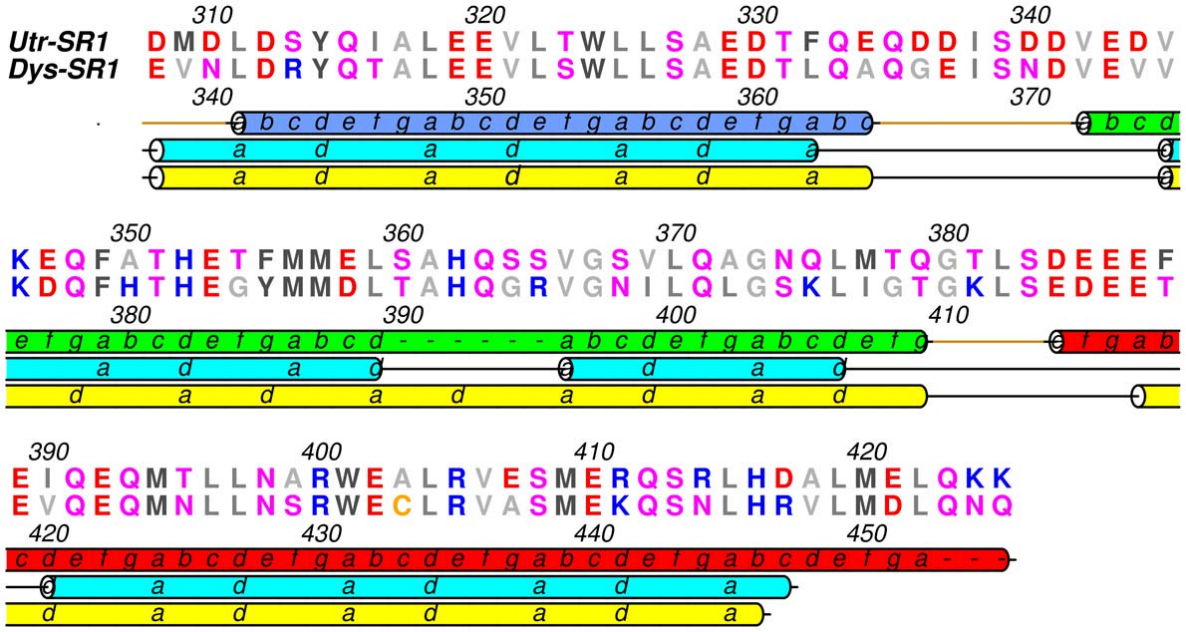
Sequence alignment of Utr-SR1 and Dys-SR1 colour-coded by amino acid conservation. The upper secondary structure cartoon shows the Utr-SR1 and Dys-SR1 crystal structure heptad repeat phasing and helix boundaries (A helix blue, B helix green and C helix red). The heptad repeat and helix boundaries for the Koenig [13] and Winder [14] sequence alignments are shown in cyan and yellow cartoon representation respectively.

Dys and Utr-SR1 adopt the canonical spectrin repeat fold comprising a triple helical bundle displaying an approximately cylindrical structure of overall dimensions ∼60 Å length and ∼9 Å radius (between backbone atoms). The three-helical bundle has a left-handed super helical twist with the three helices (A–B–C) arranged in an up-down-up topology (fig. 1). The heptad repeat sequence motif characteristic of this fold is evident in the structure with the packing of hydrophobic side chains at the ‘a’ and ‘d’ heptad repeat positions on the internal faces of the three helices forming the hydrophobic core of the triple-helical bundle structure (fig. 2). Helix A (Utr 310–334; Dys 340–364, residue number is offset by +30 for Dys-SR1 relative to Utr-SR1) is shorter than B (Utr 342–379; Dys 372–409) and C (Utr 384–423; Dys 414–453) with the triple helix structure changing to coiled-coil formed between the B and C helices after the C-terminal end of helix A, a conserved feature for spectrin repeats domains. Helices A and C are straight but helix B has an appreciable bend in the centre with the loss of canonical main chain n+4 helical hydrogen bonding for residues Utr 360–361.This bending of helix B, a conserved spectrin repeat domain structural motif [35], is stabilised by conserved side chain stacking interactions between H362 and W324 (helix A) in combination with W401 (helix C) augmenting the hydrophobic heptad repeat central domain core. These two conserved Trps are a defining feature of spectrin repeats contributing to α-spectrin domain conformational stability [48]. H362 is not strictly conserved for Utr/Dys or homologous repeats often replaced by Pro, that is also accommodated within the helix B bend. For β-spectrin the bend in helix B is proposed to act as a hinge (in addition to the A–B and B–C loops) associated with stable unfolding intermediates which were not observed when the conserved Pro, and a Gly one helical turn away (4 amino acids C-terminal), were mutated to Ala [49].

**Figure 3:**
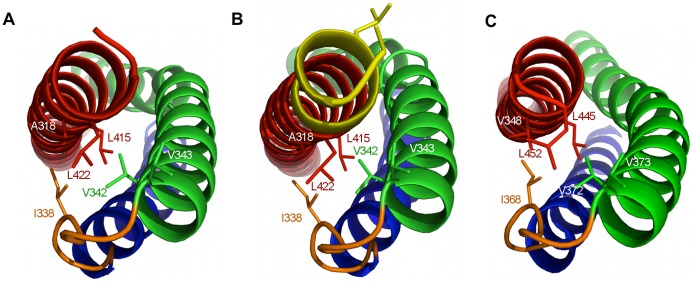
Interactions formed by the C-terminus of utrophin and dystrophin spectrin repeat one domains. Structural representations of A) Utr-SR1, B) Utr-SR1-L and C) Dys-SR1 showing the burial of hydrophobic sidechains (stick representation) on the B helix (green) and A–B loop (orange) by the C-terminus of helix C (red). The A helix is coloured blue. The extended Utr-SR1-L C-terminus and sidechains of the SR2 heptad repeat (L427, L430) are coloured yellow. The protein main-chain is depicted in ribbon representation with key side-chains shown as sticks.

Based on sequence analysis Dys repeat 1 has been previously defined as amino acids 338–446/7 [13], [14]. The compact structural domain of the Dys-SR1 (338–453) and Utr-SR1 (308–423) crystal structures is extended by six residues at the C-terminus of helix C. The Dys/Utr-SR1 structures finish with a break in the heptad repeat that coincides with a two residue gap in the Koenig sequence alignment [13], with the heptad repeat discontinuous from the C-terminus of helix C SR1 through the linker to helix A’ of SR2 although the helix itself is continuous into SR2 as evident from the extended Utr-SR1-L structure. The discontinuity of the heptad repeat at the C-terminal extension linker region is now explained as this region does not form helix-helix interactions; instead the extra helical turn and half at the C-terminus of helix C packs against the interior hydrophobic face of the SR1 A-B loop (fig. 3). The heptad repeat of Utr-SR1 helix C finishes with L422 (Dys L452) in the heptad ‘a’ position while the heptad repeat of SR2 helix A’ starts with L427 (Dys L457) in position ‘a’. From a tertiary domain structural perspective, including the linker region at the C-terminus of SR1 helix C rather than the N-terminus of helix A’ of SR2 results in a compact spectrin repeat structure for Utr/Dys-SR1. The extended C helix C-terminus is positioned to stabilise the hydrophobic face of the interior of the A–B loop; L415 (Dys L445) and L422 (Dys L452) in heptad ‘a’ repeat position and A418 (Dys V448) in ‘d’ position of the C helix pack against I338 (Dys I368), V342 (Dys V372) and V345 (Dys V375), from the A–B loop (fig. 3). The hydrophobic interface presented by the extension to helix C, provides a structural explanation of why longer spectrin repeat constructs than those suggested by sequence alignment are required for stable Utr/Dys spectrin repeats; shorter constructs would presumably leave the hydrophobic surface of the A–B loop solvent exposed reducing polypeptide stability. By homology the extended C-terminus of SR1 suggests that the compact domain structure for Dys-SR2 extends to 454–561/562 providing a rationale for the previously observed increased stability of the Dys-SR2 439–564 polypeptide relative to 439–553 and 448–556 [19], [21], [29]. A similar argument can be made with respect to the increased stability observed for an extended Dys-SR7-9 construct [30]. For β-spectrin the linker region between R8 and R9 interacts with both the A–B loop of R8 and the B’–C’ loop of R9 [35], so likewise for Utr and Dys the repeats can be considered to be overlapped by one and a half helical turns of the linker region from a structural domain perspective.

**Figure 4:**
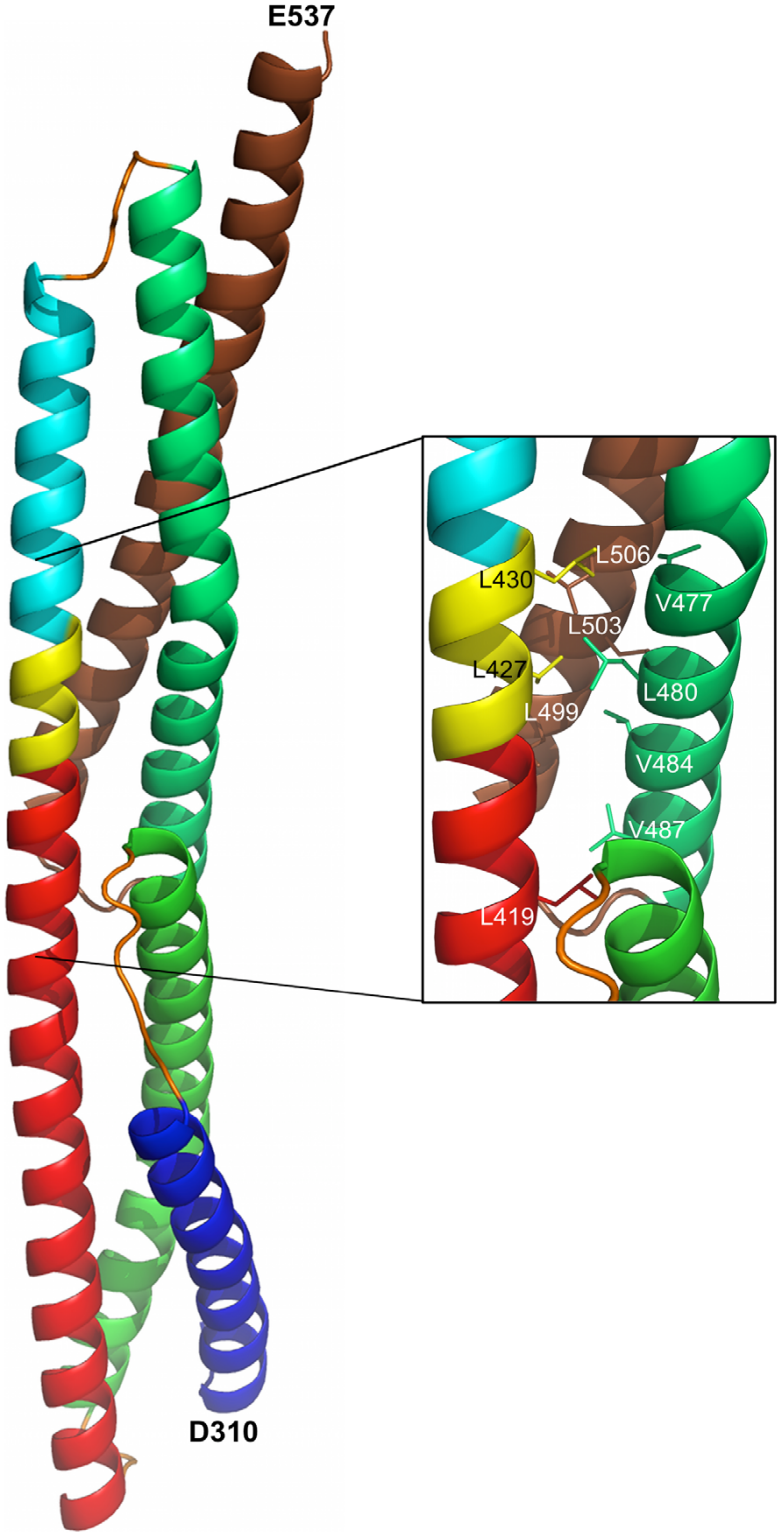
A structure for Utr-SR1-SR2 modeled from the Utr-SR1 crystal structure and a homology model for Utr-SR2. Ribbon representation of the hybrid Utr-SR1-SR2 model containing the experimentally determined Utr-SR1-L structure combined with an I-TASSER predicted model for Utr-SR2 superposed on the overlapping region (yellow). SR1 is colour coded A helix (dark blue), B helix (green), C helix (red), extended Utr-SR1-L region (yellow). SR2 is colour-coded helix A (mid-blue), helix B (green), helix C (brown). The orientation of the SR1 domain is approximately equivalent to figure 1. The close up view of the linker regions shows the heptad repeat phasing of L427 and L430 from Utr-SR1-L at the ‘a’ and ‘d’ heptad positions of helix A’ of SR2 forming coiled-coil interactions with the Utr-SR2 domain.

**Figure 5:**
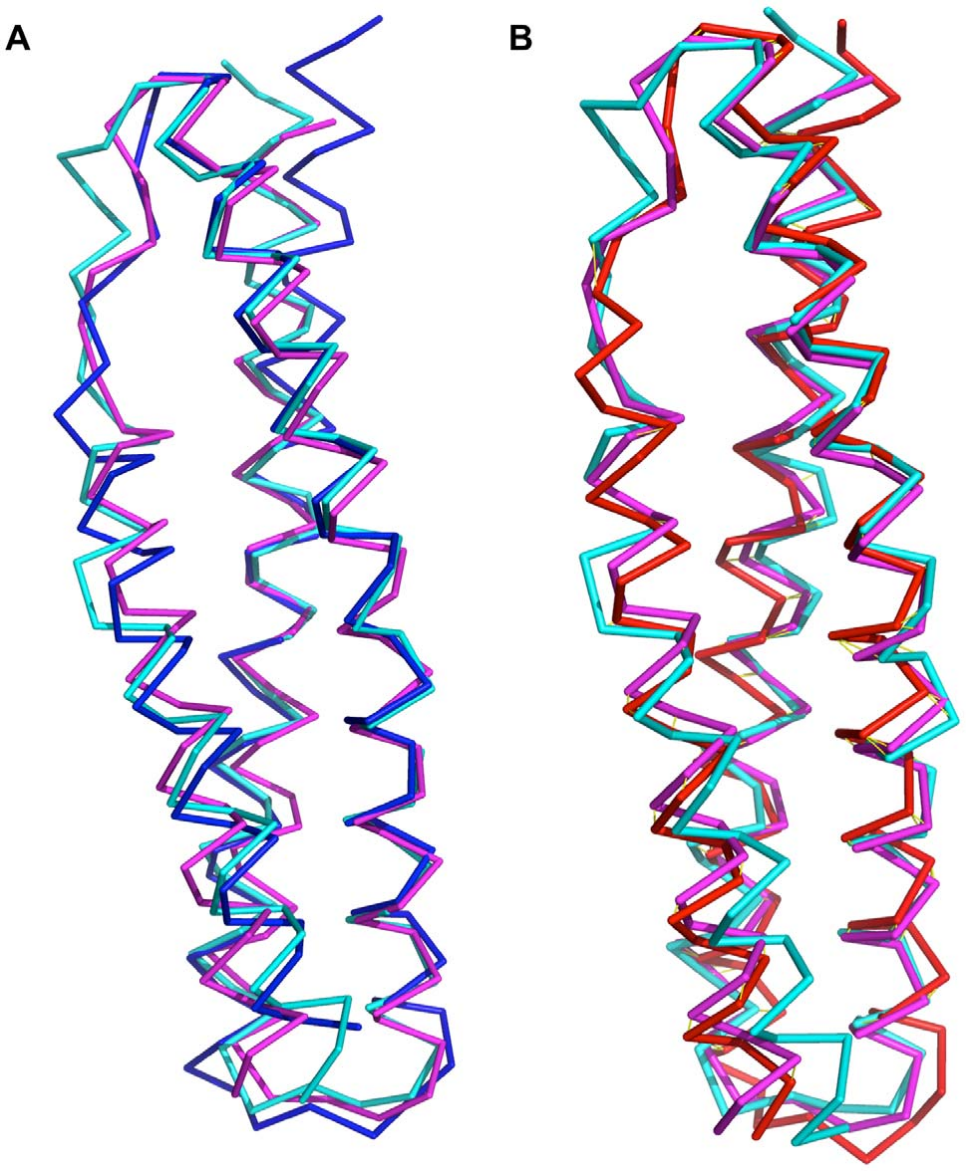
Cα backbone superpositions of Utr-SR1 and Dys-SR1 with selected homologous spectrin repeat structures. A) Utr-SR1 (blue) and B) Dys-SR1 (red) superimposed on α-actinin-2 repeat 2 (cyan, 1HCI) and α-spectrin repeat 16 (magenta, 1CUN) structures.

For the longer Utr-SR1-L structure we observe the start of the heptad repeat phasing for the next repeat domain SR2. SR2 helix A’ heptad starts with L427 at the heptad ‘a’ position and L430 in ‘d’ position both on the opposite face of the continuous A’ helix relative to the heptad ‘a’ and ‘d’ positions on SR1 helix C, inferring that the hydrophobic core of SR2, and hence the position of the SR2 B’ and C’ helices, is orientated by ∼180° rotation about the C–A’ linker helical region from the hydrophobic core of SR1 (fig. 3B). We constructed a hybrid model for Utr-SR1-SR2 by superposing a model for UTR-SR2, predicted by I-TASSER [50], onto the linker and SR2-helix A’ region of the Utr-SR1-L structure (fig. 4). This approach positions the B’ and C’ helices of SR2 against the opposite face of the continuous C-A’ helix differently to α-actinin and spectrin two repeat structures. The very C-terminus of Utr-SR1-L, corresponding to the N-terminus of SR2 helix A’, has rotated about the C-A’ helix axis, relative to homologous structures, forming an anti-parallel inter-helix interaction with a crystallographically-related molecule. It is likely that this rotation is a result of crystal packing rather than a reorientation of SR2 relative to SR1 although variation in domain orientation is observed for this protein family [34], [40]; a complete Dys/Utr-SR1-SR2 structure is required to conclusively define the inter-domain orientation. Differences in spectrin repeat orientation between Dys/Utr compared to α-actinin or spectrin may not be completely unexpected since Dys/Utr repeats are not restrained by the formation of antiparallel dimers that occurs for α-actinin and spectrin. The structures of Dys-SR1 and Utr-SR1 are both similar to individual α-actinin, spectrin and plectin spectrin repeats (Cα rmsd 1.5–2.4 Å; table 2) with differences occurring mainly in the A–B and B–C loop regions (fig. 5).

**Table 2:** Comparison of Utr-SR1 and Dys-SR1 to selected spectrin repeat domain structures.

Spectrin repeat domain	PDB ID	Utr-SR1 rmsd Å (Cα)	Utr-SR1% seq. id	Dys-SR1 rmsd Å (Cα)	Dys-SR1% seq. id
Chicken brain α-spectrin repeat 16	1CUN	1.7 (111)	22	1.5 (110)	17
Human skeletal muscle α-actinin-2 repeat 2	1HCI	1.7 (111)	18	1.6 (112)	21
Human erythrocyte α-spectrin repeat 1	3LBX	1.8 (109)	18	1.7 (110)	18
Human erythrocyte β-spectrin repeat 14	3F57	1.8 (111)	20	1.8 (112)	18
Human erythrocyte β-spectrin repeat 8	1S35	2.1 (111)	18	1.7 (107)	18
Chicken brain α-spectrin repeat 15	1U4Q	1.8 (111)	16	1.8 (112)	16
Chicken brain β-spectrin repeat 14	3EDV	1.8 (111)	18	2.0 (112)	19
Human plectin repeat 3	3PDY	2.4 (107)	14	2.1 (106)	14

The root mean squared differences (rmsd) in Å between repeat domain structures are reported. The number of Cα atoms used in calculating the rmsd for each pair-wise comparison is included in brackets. ‘% seq. id’ is % sequence identity.

**Figure 6:**
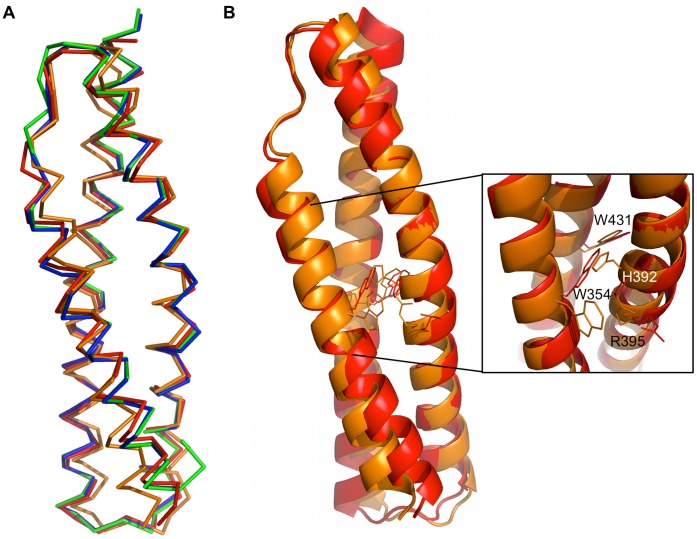
Comparison of the Utr-SR1 and Dys-SR1 structures. A) Cα backbone superposition of the two Utr-SR1 and the two Dys-SR1 molecules of their respective crystallographic asymmetric units: Utr-SR1; molecule A (blue), molecule B (green). Dys-SR1; molecule A (red), molecule B (orange). B) Superposition of Dys-SR1 molecules A (red) and B (orange) highlighting the difference in W354 and H392 sidechain rotamers involved in the usually conserved spectrin repeat core stacking interaction.

Superimposing the two Utr-SR1 molecules (A and B) in the asymmetric unit with each other shows they are very similar with rmsd of 0.5 Å (113 Cα) and the only obvious difference a rigid body shift (∼1 Å) of the A–B loop (residues 337–341) (fig. 6A). Analysis of molecular packing in the Utr-SR1 crystal revealed an interaction predicted to be relevant with respect to oligomerisation burying 1260 Å^2^ surface area between monomers A and B. However size exclusion chromatography data for Utr/Dys-SR1, as well as previous studies [16], show that Utr (and Dys) are monomeric suggesting that the degree of association observed in the crystal is most likely owing to crystal packing and the non-globular nature of the SR tertiary structure.

The two Dys-SR1 molecules in the asymmetric unit superimpose with rmsd 1.5 Å (112 Cα) (fig. 6A). Comparing the structure of Dys-SR1 with Utr-SR1 results in rmsds of 0.8–1.9 Å (112 Cα) depending on which of the four molecules in the asymmetric unit of the two crystals are superimposed in a pair-wise manner. One Dys-SR1 molecule (molecule B; Dys-SR1B) is the most different; the conserved W354, in the centre of helix A, has well defined electron density showing that it adopts the less common χ_1_ −99° sidechain rotamer instead of the more frequent χ_1_ 180° conformation, found in Dys-SR1 molecule A and both Utr-SR1 molecules (fig. 6B). Instead of forming the usually conserved interactions with H392 (heptad ‘d’ position, helix B) and W431 (‘a’, helix C) Dys-SR1B W354 stacks against R395, one helical turn distant from 392, and is associated with both a twisting of the bend region in the centre of helix B and a crystal contact (M387 distance 3.5 Å) from a crystallographically-related molecule. This disruption of sidechain interactions relaxes the Dys-SR1B main chain so that canonical helical main chain hydrogen bonds are formed in contrast to Dys-SR1 monomer A and Utr-SR1. This rearrangement appears to be caused by crystal packing effects but it is noteworthy that this conserved Trp interaction within the three-helix bundle core does display plasticity that may have implications for repeat domain mechanical and unfolding properties. Dys-SR1A and Utr-SR1 (A & B) form the more typical conserved interaction between W354/W324 with helix B H392/H362 and helix C W431/W401. Dys-SR1 crystallisation has been aided by a disulphide bond between C433 from each molecule in the asymmetric unit (fig. S1). Dimerisation in solution could be reversed by reducing the disulphide bond with the addition of 5 mM DTT, as analysed by analytical size exclusion chromatography (fig. S2), but reduced Dys-SR1 failed to crystallise. Crystallisation via symmetrical homodimerisation through disulphide bond formation has been previously documented as an experimental strategy [51]. Curiously the Utr-SR1 (and Utr-SR1-L) structures are the first single spectrin repeat structures determined by x-ray crystallography in the Protein Data Bank; the single drosophila α-spectrin repeat 14 structure has been determined [31] but crystallizes in a domain-swapped dimer conformation rather than the isolated three-helix bundle fold and Dys-SR1 is disulphide linked. However, it is not possible to conclude whether the increased proportion of multiple repeat structures crystallised reflects difficulties in determining single repeat domain boundaries suitable for crystallisation, inherent structural properties of single repeats, or simply a preference for the multiple repeat constructs that have been experimentally tested.

### Utr-SR1 and Dys-SR1 have no Affinity for F-actin

**Figure 7:**
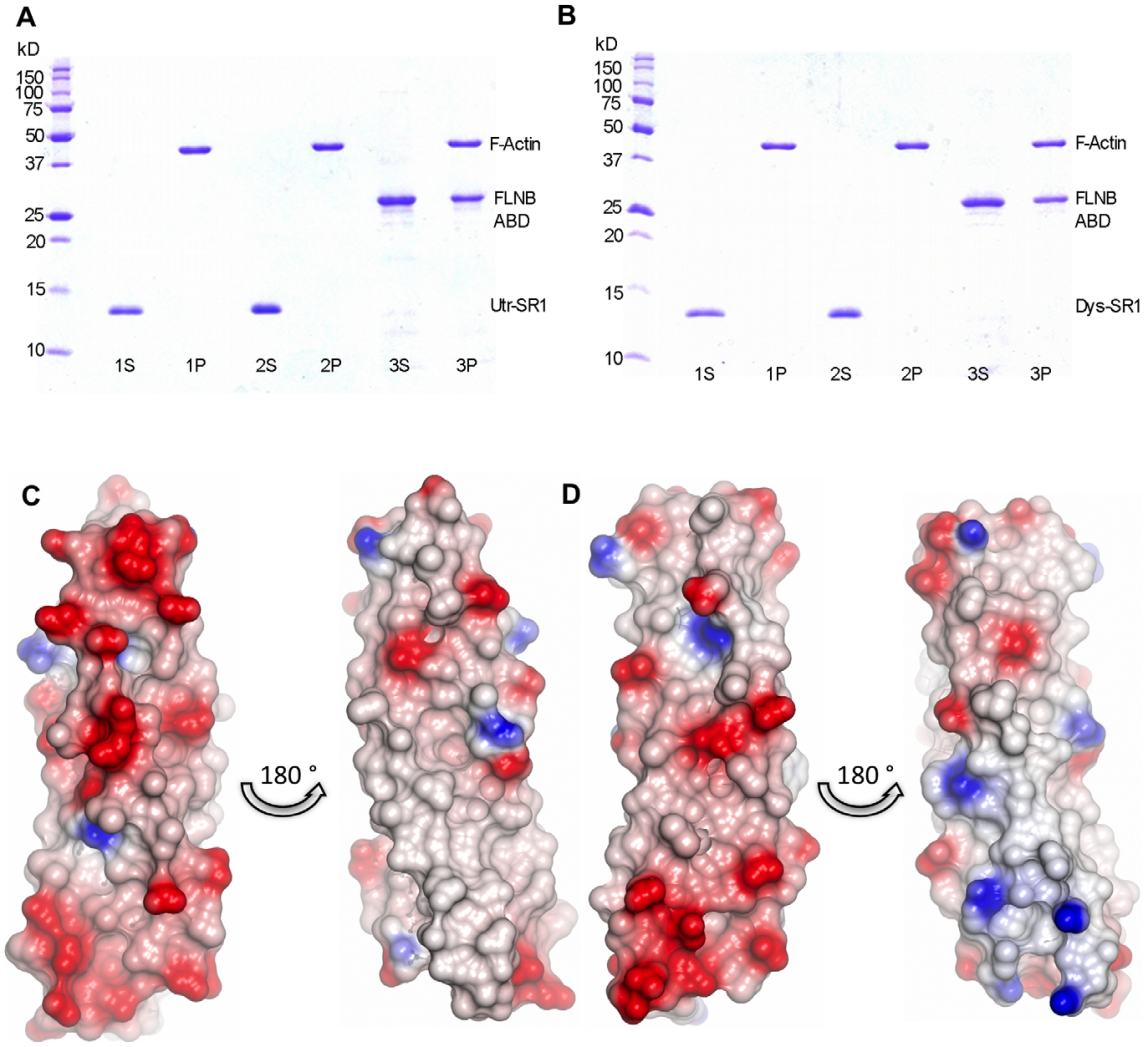
Dys-SR1 and Utr-SR1 exhibit no F-actin binding activity. A) Utr-SR1 and B) Dys-SR1 F-actin binding co-sedimentation assay SDS-PAGE gels. Lane 1; MW markers. Lanes 2–6; S and P are supernatant and pellet fractions; actin concentration is 8 µM. A) 1S, 1P; 20 µM Utr-SR1 & F-actin. 2S, 2P; 40 µM Utr-SR1 & F-actin. 3S, 3P; F-actin & 20 µM Filamin B (FLNB) actin binding domain (ABD) as a positive control found in the pellet fraction bound to F-actin. B) 1S, 1P; 20 µM Dys-SR1 & F-actin. 2S, 2P; 40 µM Dys-SR1 & F-actin. 3S, 3P; 20 µM FLNB-ABD & 8 µM F-actin. Utr-SR1 and Dys-SR1 are found in the supernatant not associated with F-actin (which is in the pellet). Empty gel lanes are left between each sample to prevent any adjacent band overlap. C) Utr-SR1 and D) Dys-SR1 structural electrostatic surface representations. The left hand views of each pair have an orientation approximately equivalent to figure 1, the right hand views are rotated ∼180° around the vertical axis. The surface has been colour coded by electrostatic potential, red −0.5 V to blue 0.5 V.

Electron micrographic image reconstruction of F-actin filaments decorated with Utr416, a construct containing the Utr ABD and a truncated SR1 (defined by previous sequence alignment) showed density for SR1 associated laterally with F-actin [52]. This observation, coupled with the identification of an F-actin binding site within the N-terminal ten utrophin spectrin repeats [24], suggested that SR1 might have intrinsic affinity for F-actin. However F-actin co-sedimentation assays showed no affinity between Utr/Dys-SR1 domains for F-actin in the absence of the ABD at the concentrations investigated (figs. 7A & 7B). Sequence analysis predicts the isoelectric points of Utr/Dys-SR1 domains to be ∼4, the most acidic of all the Utr/Dys spectrin repeats. Analysis of electrostatic surface representations of the Utr/Dys-SR1 structures highlights one particular acidic surface running along the faces of helices A and B, including the Glu-rich B-C loop (figs. 7C & 7D). This negative electrostatic character correlates with a lack of affinity with acidic F-actin for the isolated Dys/Utr-SR1 in contrast to F-actin binding Dys basic spectrin repeats [53]. Mapping sequence conservation to the Utr-SR1 structure using ConSurf [54] highlights a strongly conserved groove on the B-C face of SR1 (fig. S3). This groove has conservation higher than can be accounted for by the heptad repeat inherent to the spectrin repeat structure; conserved regions such as this are often associated with protein function [54] such as interaction binding sites, though no binding partners specific for SR1 have been identified to date.

## Discussion

Despite the similarities of full-length Dys and Utr at the sequence level, and in terms of their broader functional properties, functional differences have been observed. For example Dys and Utr bind F-actin with equivalent binding affinities yet exhibit differences in the mechanism of actin binding. Dys has two F-actin binding sites; the ABD and repeats 11–17 in contrast to one continuous site for Utr (ABD-repeat 10) [55]. These differences in spectrin repeat properties could potentially have important consequences if therapeutic strategies of replacing defective or missing Dys with *UTR/DYS* mini transgenes that omit certain spectrin repeats [42], [46] are to be explored in a clinical environment. It had been previously predicted from sequence analysis that Dys/Utr spectrin repeats could fold into three helix bundle structures [56], that we now show in atomic detail. The tertiary structure of the Utr/Dys spectrin repeat is now characterised defining the exact boundaries of the helices, the nature of the kink in the centre of helix B and the C-terminal repeat linker region with respect to previous predictions. The Dys/Utr spectrin repeat is structurally defined here as the most globular-like domain extending the sequence repeat by six residues for one and half helical turns. Hence the sequence repeat can be considered overlapped with respect to this compact domain structure. How far this conclusion can be extended throughout the entire Dys/Utr rod region still remains to be absolutely determined as Dys and Utr spectrin repeats are not as regular as those from α/β-spectrin and α-actinin, containing additional interruptions and insertions/deletions that disrupt the heptad phasing (e.g. repeats 4, 5, 16). Additionally the Utr/Dys first repeat may be atypical at its N-terminus because it has no preceding repeat. Further structural characterization of multiple Dys and Utr repeat domain constructs is required.

## Materials and Methods

### Expression and Purification of Utrophin and Dystrophin Spectrin Repeat Proteins

Utr-SR1 cDNA (rat, amino acids 308 to 425) was prepared by PCR from UT11 [57] as template and ligated as a BamHI/XhoI digested fragment into pProEXHtb. *E. coli* BL21(DE3) were transformed with this construct and grown in LB media plus 0.1 mg/ml ampicillin at 37°C to A_600 nm_ 0.5–0.6 when isopropyl-β-D-thiogalactoside was added (0.1 mM final concentration) to induce protein expression with the cells grown further at 25°C overnight. The cells were harvested by centrifugation, washed in PBS and frozen. Cell pellets were subsequently thawed and resuspended in 20 mM Tris (pH 8.0), 120 mM NaCl, 1 mM β-mercaptoethanol, 5 mM imidazole, Complete™ EDTA-free protease inhibitors (Roche) and lysozyme (1 mg/ml), and then incubated on ice for 20 minutes. Cells were lysed by passage twice through a French press (4000 psi) and the lysate centrifuged at 17 000 g for 20 mins. The lysis supernatant was applied to Ni^2+^-NTA resin, pre-equilibrated in 20 mM Tris pH 8.0, 120 mM NaCl, 1 mM β-mercaptoethanol, 5 mM imidazole, and washed extensively with the same buffer. Utr-SR1 was eluted with wash buffer made to 50–100 mM imidazole concentration with fraction purity confirmed by SDS-PAGE. The His-tag was removed by digestion with recombinant tobacco etch virus protease overnight at 4°C with the resulting His-tag and the protease subsequently separated by reapplication to Ni^2+^-NTA. Utr-SR1 was dialyzed against 20 mM Tris pH 8.0, 50 mM NaCl and injected onto a Superdex 75 HR 30/10 size exclusion column. Utr-SR1 eluted as a single peak with profile consistent with a monomer. Utr-SR1-L was initially expressed and purified as Utr-SR1-SR2 (residues 308–537) by a similar protocol. The Dys-SR1 construct (human, residues 338–456) was prepared in an equivalent manner with similar protein expression and purification protocols except the buffer was PBS and for Dys-SR1 a second size exclusion chromatography peak of elution volume consistent with a dimeric species was observed. This dimer species could be converted to monomer by reduction with 5 mM DTT consistent with a disulfide-linked dimer as observed in the Dys-SR1 crystal structure.

### Crystallisation and Structure Determination

Initial Utr-SR1 crystals were obtained at 4°C by mixing 0.5 µl Utr-SR1 at a concentration of 8.2 mg/ml with equal volume mother liquor in sitting drops using 96-well plates with 100 µl reservoir well volumes of Hampton Research Crystal Screens I and II. Optimization of the crystallisation conditions proceeded by hanging-drop vapour diffusion at 4°C with the crystallisation mother liquor solution containing 0.2 M MgCl_2_ and 25% (w/v) PEG 4000 in 0.1 M Tris (pH 8.5). Dys-SR1 at 7.6 mg/ml in PBS buffer crystallized from 2.0 M (NH_4_)_2_SO_4_ in 0.1 M Tris (pH 8.5) at room temperature. Utr-SR1-L was crystallized from 0.1 M sodium cacodylate (pH 6.5), 20% (w/v) PEG 8000, 0.2 M magnesium acetate at room temperature.

The Utr-SR1 crystals were orthorhombic with spacegroup P2_1_2_1_2_1_ containing two molecules in the asymmetric unit corresponding to a solvent content of ∼40%. X-ray diffraction data (wavelength 1.5418 Å), collected at 120°K, were processed to an outer resolution of 1.95 Å. The structure of Utr-SR1 was solved by molecular replacement with PHASER [58] using a search model derived from the crystal structure of the α-actinin spectrin repeats [36]. The position of the two molecules in the asymmetric unit was clearly distinguishable from the noise of the rotation and translation functions confirming the choice of space group. The structure was refined with REFMAC [59] and PHENIX [60] with model rebuilding in COOT [61] using maps calculated from refinement coefficients. Positive difference density was visible for side chains that were not included in the search model. Solvent molecules were added to the model if a positive 3 sigma peak existed in the Fo-Fc difference map in a position in which chemically sensible hydrogen bonds could be made. A total of 206 solvent atoms were included. The model was refined by cycles of rebuilding followed by maximum-likelihood refinement finishing with TLS refinement in the final stages [62]. The refinement converged to yield a final model with an R-factor of 0.200 for all data 36 Å to 1.95 Å and free R-factor of 0.235 (calculated from 5% reflections omitted from refinement). Further model statistics are presented in table 1. The Dys-SR1 crystals have spacegroup P3_2_21 with two molecules present in the asymmetric unit corresponding to a solvent content of ∼38%. X-ray diffraction data were processed to an outer resolution of 2.3 Å. The Dys-SR1 structure was determined by molecular replacement using Utr-SR1 as a search model using similar protocols. This crystal contained a minor twin domain (−h, −k, l) of 8%. Dys-SR1 was rebuilt and refined in a similar manner to Utr-SR1 to yield a final model with R-factor 0.193, R_free_ 0.260 (calculated from 5% reflections omitted from refinement). The Utr-SR1-L structure was determined using the isomorphous Utr-SR1 as an initial model. The C-terminus was extended by manual model building into visible electron density and refined with REFMAC to yield a final model with R-factor 0.201, R_free_ 0.268 (calculated from 5% reflections omitted from refinement). Figures were prepared with PyMOL [63] and CCP4 mg [64]. Sequences were aligned with ClustalX [65] and displayed with Aline [66]. Structural comparisons were conducted with DALI [67] and surface analysis with PISA [68].

### Actin Binding Assay

The binding of Utr/Dys-SR1 to F-actin was analyzed by a cosedimentation assay, conducted under similar conditions as described [69] in a buffer of 20 mM Tris-HCl pH 8.0, 120 mM NaCl, 2 mM MgCl_2_, 1 mM Na_2_ATP, 5 mM DTT. Briefly, actin from rabbit skeletal muscle (Cat #AKL99, Cytoskeleton, Inc.) was polymerised into F-actin by the addition of 1 mM ATP, 2 mM MgCl_2_ and 50 mM KCl with incubation at 25°C for 60 minutes, then added to each reaction tube for a final concentration of 8.2 µM. Two concentrations (20 and 40 µM) of Utr-SR1 or Dys-SR1 were incubated with the F-actin in assay buffer (50 mM Tris-HCl pH 8.0, 120 mM NaCl, 5 mM DTT) for 1 hour at 25°C. The fractionation of the Utr-SR1/Dys-SR1 into the supernatant, with none in the pellet fraction associated with F-actin, after ultracentrifugation at 214, 000 x *g* for 30 minutes at 25°C revealed no detectable F-actin binding activity. Equal volumes of supernatant and pellet fractions were visualized using 12% SDS-PAGE and Coomassie Brilliant Blue staining. The Filamin B ABD was used as a positive control for F-actin binding [70] and is detected in the pellet.

## Supporting Information

Figure S1 **The two Dys-SR1 molecules comprising the crystallographic asymmetric unit in ribbon representation.** The inter-molecular disulfide bond between C433 from each molecule is shown in stick representation.
(TIF)

Figure S2 **Utr-SR1 and Dys-SR1 size exclusion chromatography.** A) Utr-SR1 (blue) and B) Dys-SR1 (red) superdex 75 size exclusion chromatographs showing the higher Mw dimer peak present for Dys-SR1 and not Utr-SR1.
(TIF)

Figure S3 **Space-filling representation of the Utr-SR1 and Dys-SR1 structures colour-coded by ConSurf** [**54**] **sequence conservation (colour ramped from purple most conserved, to blue most variable, with white intermediate).** LHS representation is orientated approximately equivalent to figure 1 and the RHS representation is rotated from the left view by 180° about the vertical axis.
(TIF)
