# Supplementary figures and images for: The Crystal Structures of Dystrophin and Utrophin Spectrin Repeats: Implications for Domain Boundaries
Source: PLoS One. 2012 Jul 20;7(7):e40066. doi: 10.1371/journal.pone.0040066 (PMC3401230; doi:10.1371/journal.pone.0040066)

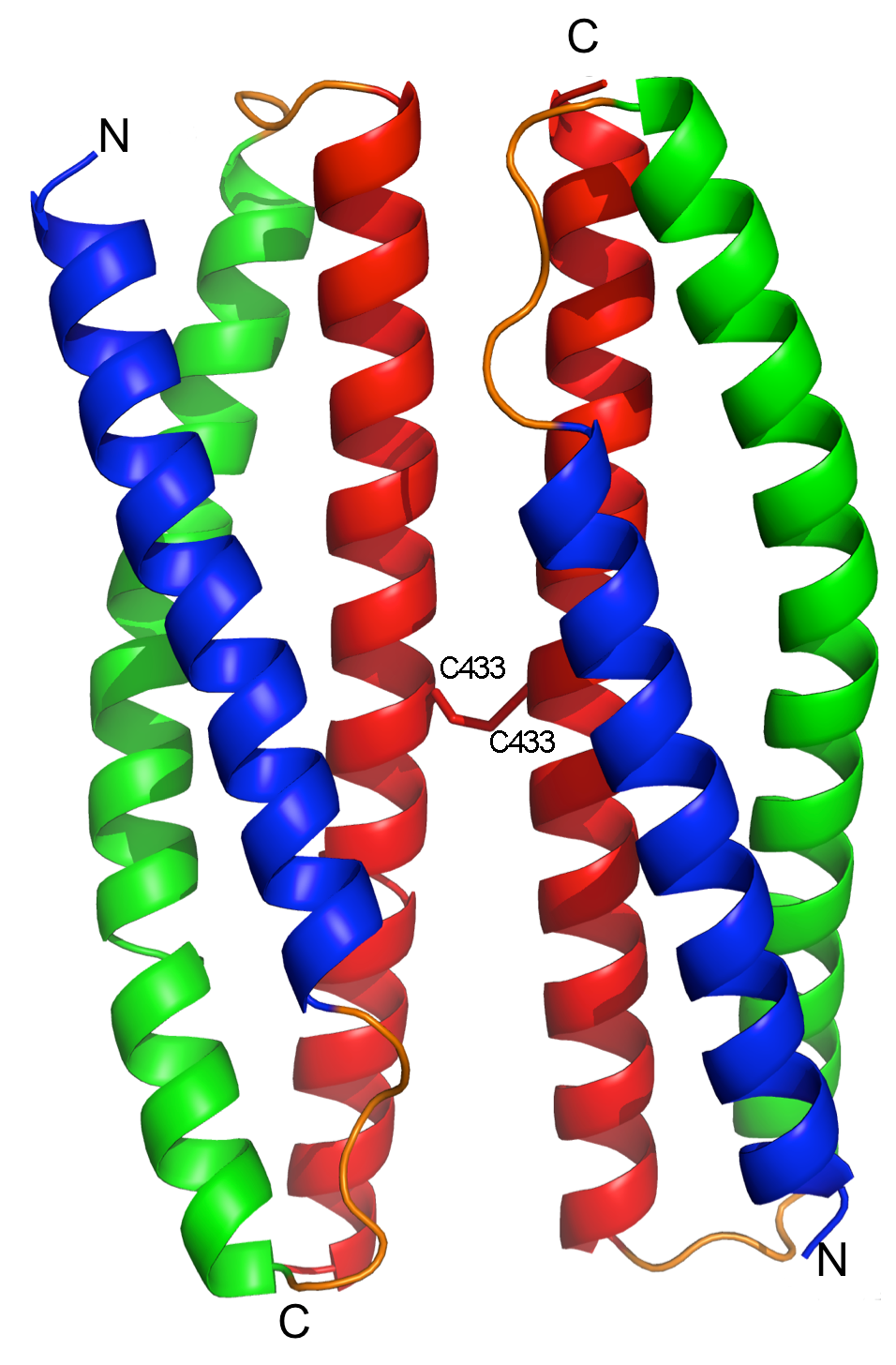

Supplement: Figure S1 — The two Dys-SR1 molecules comprising the crystallographic asymmetric unit in ribbon representation. The inter-molecular disulfide bond between C433 from each molecule is shown in stick representation. (TIF) [file pone.0040066.s001.tif]

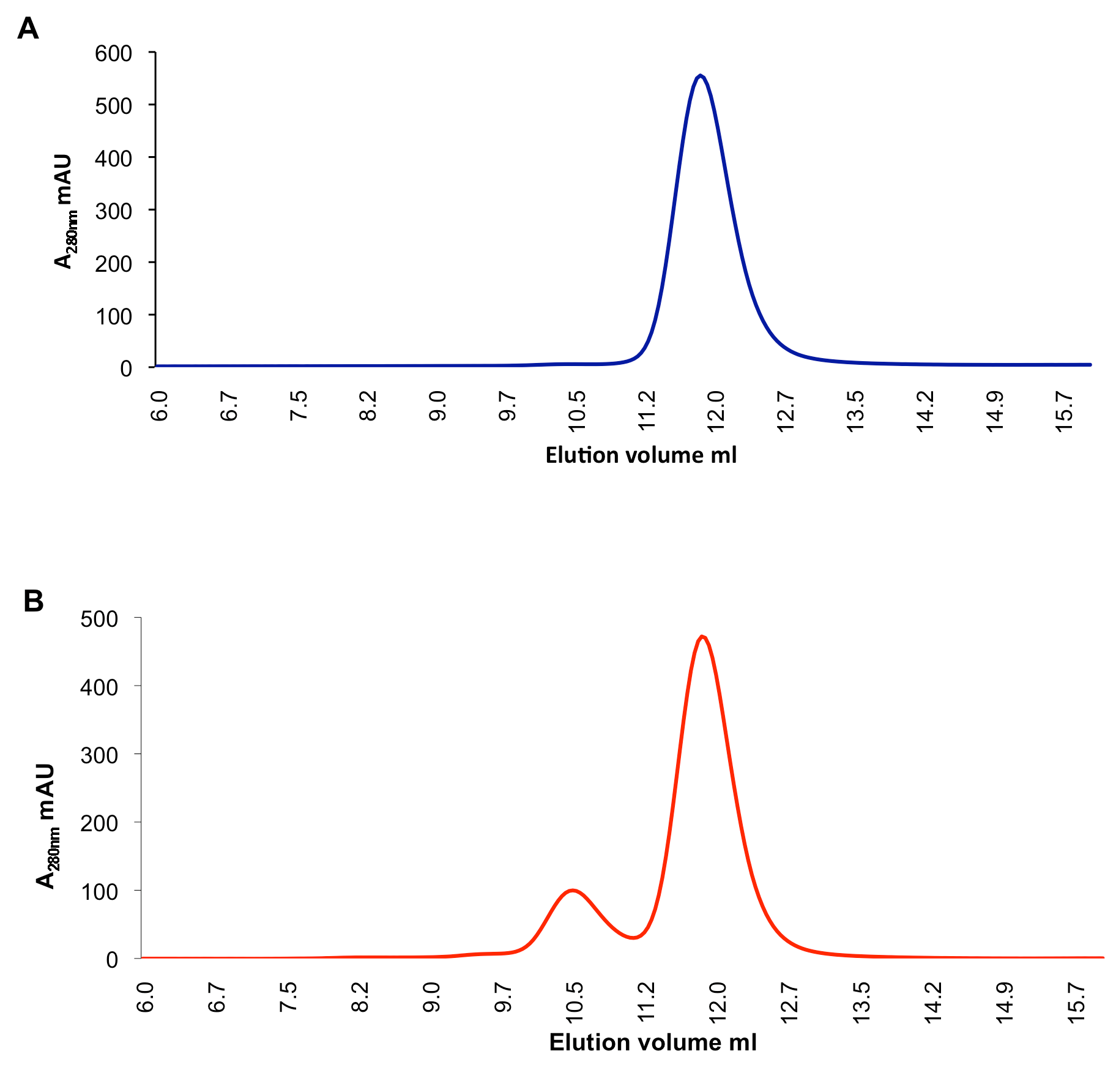

Supplement: Figure S2 — Utr-SR1 and Dys-SR1 size exclusion chromatography. A) Utr-SR1 (blue) and B) Dys-SR1 (red) superdex 75 size exclusion chromatographs showing the higher Mw dimer peak present for Dys-SR1 and not Utr-SR1. (TIF) [file pone.0040066.s002.tif]

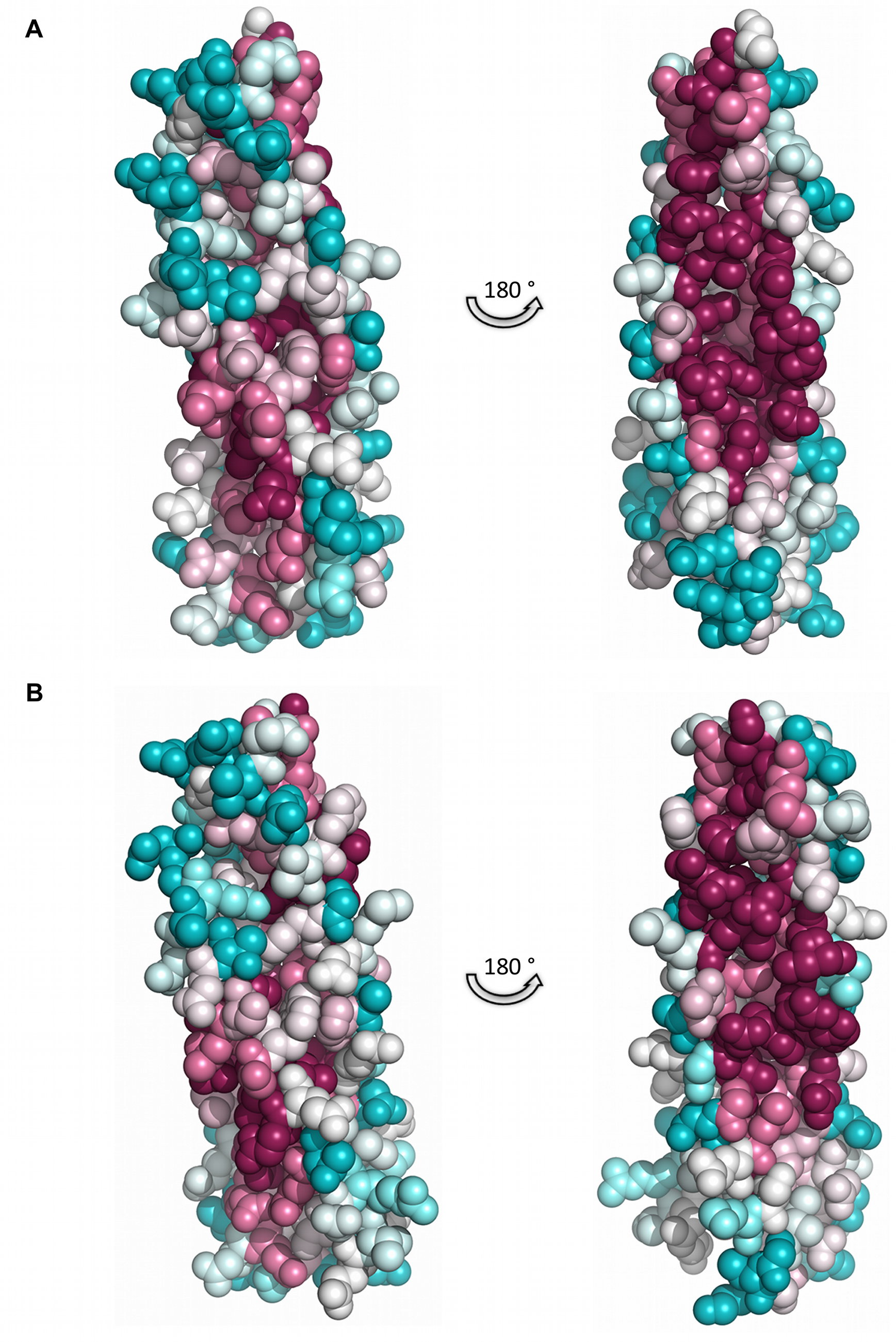

Supplement: Figure S3 — Space-filling representation of the Utr-SR1 and Dys-SR1 structures colour-coded by ConSurf [54] sequence conservation (colour ramped from purple most conserved, to blue most variable, with white intermediate). LHS representation is orientated approximately equivalent to figure 1 and the RHS representation is rotated from the left view by 180° about the vertical axis. (TIF) [file pone.0040066.s003.tif]
